# Is Elevated Choline on Magnetic Resonance Spectroscopy a Reliable Marker of Breast Lesion Malignancy?

**DOI:** 10.3389/fonc.2021.610354

**Published:** 2021-09-10

**Authors:** Natasa Prvulovic Bunovic, Olivera Sveljo, Dusko Kozic, Jasmina Boban

**Affiliations:** ^1^Department of Radiology, Faculty of Medicine, University of Novi Sad, Novi Sad, Serbia; ^2^Center for Diagnostic Imaging, Oncology Institute of Vojvodina, Sremska Kamenica, Serbia; ^3^Department for Telecommunications and Signal Processing, Faculty of Technical Sciences, University of Novi Sad, Novi Sad, Serbia

**Keywords:** magnetic resonance imaging, breast, breast neoplasms, magnetic resonance spectroscopy, biomarkers

## Abstract

**Background:**

Contemporary magnetic resonance imaging (MRI) of the breast represents a powerful diagnostic modality for cancer detection, with excellent sensitivity and high specificity. Magnetic resonance spectroscopy (MRS) is being explored as an additional tool for improving specificity in breast cancer detection, using multiparametric MRI. The aim of this study was to examine the possibility of 1H-MRS to discriminate malignant from benign breast lesions, using elevated choline (Cho) peak as an imaging biomarker.

**Methods:**

A total of 60 patients were included in this prospective study: 30 with malignant (average age, 55.2 years; average lesion size, 35 mm) and 30 with benign breast lesions (average age, 44.8 years; average lesion size, 20 mm), who underwent multiparametric MRI with multivoxel 3D ^1^H-MRS on a 1.5-T scanner in a 3-year period. Three patients with benign breast lesions were excluded from the study. All lesions were histologically verified. Peaks identified on ^1^H-MRS were lipid (0.9, 2.3, 2.8, and 5.2 ppm), choline (3.2 ppm), and water peaks (4.7 ppm). Sensitivity and specificity, as well as positive and negative predictive values, were defined using ROC curves. Cohen’s Kappa test of inter-test reliability was performed [testing the agreement between ^1^H-MRS and histologic finding, and ^1^H-MRS and MR mammography (MRM)].

**Results:**

Choline peak was elevated in 24/30 malignant lesions and in 20/27 benign breast lesions. The sensitivity of ^1^H-MRS was 0.8, specificity was 0.741, positive predictive value was 0.774, and negative predictive value was 0.769. Area under ROC was 0.77 (CI 0.640–0.871). Inter-test reliability between ^1^H-MRS and histologic finding was 0.543 (moderate agreement) and that between ^1^H-MRS and MRM was 0.573 (moderate agreement). False-negative findings were most frequently observed in invasive lobular cancers, while false-positive findings were most frequently observed in adenoid fibroadenomas.

**Conclusion:**

Although elevation of the choline peak has a good sensitivity and specificity in breast cancer detection, both are significantly lower than those of multiparametric MRM. Inclusion of spectra located on tumor margins as well as analysis of lipid peaks could aid both sensitivity and specificity. An important ratio of false-positive and false-negative findings in specific types of breast lesions (lobular cancer and adenoid fibroadenoma) suggests interpreting these lesions with a caveat.

## Introduction

Breast cancer is one of the most prevalent cancers in the female population in developed countries, with over 268,800 new cases of invasive breast cancers estimated in 2019 and with a tendency of moving towards younger age groups (<50 years) (1). Magnetic resonance imaging (MRI) is a powerful and reliable imaging technique that became an important milestone in breast cancer imaging with the introduction of dynamic contrast-enhanced MRI (DCE-MRI). This method has an excellent sensitivity—reaching 100%—and high specificity—ranging from 71% to 97% (2, 3). It led to the introduction of DCE-MRI as a screening tool for women at high risk of breast cancer (20%–25% estimated lifetime risk) in 2007 (4). Advanced imaging techniques, such as diffusion-weighted imaging (DWI) and magnetic resonance spectroscopy (MRS), have been added to the research and clinical protocols recently with the aim to increase the specificity of the DCE-MRI.

^1^H-MRS exhibits a typical peak at 3.2 ppm that is referred to as total-choline containing compounds (tCho), since it consists of several peaks (including phosphocholine, glycerophosphocholine, and free choline) *in vivo* observed as a single peak (5). It is suggested that intracellular phosphocholine is increased in malignant lesions, in the concentration that positively correlates with a higher grade of the lesion (6, 7). This finding directed the research towards detection and quantification of tCho in the breast lesions targeted to determine their malignant potential.

The main aim of the study was to examine the potential of *in vivo* ^1^H-MRS to discriminate malignant from benign breast lesions, using elevated choline (Cho) peak as an imaging biomarker. Additional aims of the study were to correlate the size and histological type of the breast lesion with the findings on ^1^H-MRS, as well as to compare the specificity and sensitivity of magnetic resonance mammography (MRM) alone to ^1^H-MRS alone on this study population.

## Methods

### Study Population

A total of 60 female patients with single breast lesions were included in this prospective, cross-sectional study in a 3-year period (July 2015–September 2018): 30 patients with malignant breast lesions, average age 55.2 years (range 39–88), and 30 patients with benign breast lesions, average age 44.8 years (range 23–71). Demographic and clinical data of the patients are given in **Table 1**. All patients had clinically palpable lesions (53 patients had mass lesions and 7 patients had non-mass-like lesions). All lesions were histologically verified, after core biopsy or surgical biopsy. Majority of the patients (54) underwent image-guided core biopsy, and 6 patients underwent surgical biopsy after the appropriate imaging.

**Table 1:** Demographic and clinical data of the patients.

	Malignant lesions	Benign lesions
**Age** [mean (range)]	55.2 (39–88)	44.8 (23–71)
**Menarche** [age, mean (range)]	12.1 (11–16)	12.88 (10–16)
**Menopause** [age, mean (range)]	51.32 (41–56)	50.6 (42–55)
**Lactation** (% of positive answer)	35.53%	37.78%
**Hereditary breast cancer** (% of positive family history)	<5%	<5%

Inclusion criteria were age over 18, female gender, presence of the symptomatic/palpable lesion in the breast, available mammography and/or ultrasound verifying the presence of breast lesion ≥10 mm in size, and classification of the lesions as suspicious based on the Breast Imaging Reporting and Data System (BI-RADS)—as BI-RADS 3, 4 and 5 category.

Exclusion criteria were lesions classified as not suspicious according to the BI-RADS system (categories 1 and 2), multifocal/multicentric breast lesions (these patients were excluded to avoid multiple biopsies of the breast in the same patient to obtain the histological information), patients with a known primary cancer other than breast cancer, patients with metastatic disease, contraindications for MRI, technically inadequate quality of spectra, and/or multiparametric MRM.

Three patients with benign breast lesions were excluded from the study due to low quality of the obtained spectra. After completion of the diagnostic protocol, all lesions were histologically examined, following core or open surgical biopsy.

The study was approved by the institutional ethical committee, and all the patients signed an informed consent to participate in the study, according to the Declaration of Helsinki.

### Breast Imaging

For patients in the reproductive period, MRM was performed in the first half of the menstrual cycle (between the 5th and 12th day). All MR imaging was performed in the 4 weeks following initial diagnostic method (digital mammography and/or ultrasound). Lesions that were detected on MRM were classified according to the BI-RADS (8).

Magnetic resonance mammography was performed on a 1.5-T clinical scanner (Avatno, Siemens, Erlangen, Germany) using a bilateral dedicated phased-array breast coil, following the standard dynamic contrast enhances (DCE) protocol, consisting of non-fat-suppressed T2-weighted turbo spin echo axial [time of repetition (TR)/time of echo (TE) 4600 ms/90 ms, field of view (FOV) 330, matrix 192 × 256, slice thickness 4 mm], STIR (short tau inversion recovery) sagittal, and 3D T1-weighted FLASH (Fast Low Angle SHot) axial (TR/TE 4.2 ms/1.6 ms, FOV 340 × 340, slice thickness 2 mm) tomograms in a dynamic contrast-enhanced manner [one precontrast and seven postcontrast acquisitions at 2-s intervals, following contrast administration at a dose of 0.1 mmol/kg at a rate of 2.5 ml/s (followed by 20 ml saline bolus)]. After standard protocol, 3D multivoxel ^1^H-MRS was performed by placing the voxel grid, size 4 × 4 × 4cm in the lesion and its surroundings; the size of a single voxel was 1 ml; the number of voxels analyzed depended on the lesion that was observed. Parameters of the sequence were TR/TE 200 ms/288 ms. The grid was manually placed by an experienced breast radiologist (with over 10 years experience in breast imaging) ensuring that the lesion is in the center of the voxel grid. Voxels located in the viable part of the tumor with the most intensive contrast enhancement were analyzed. Peaks of lipid, water, and choline (Cho) were detected at the spectroscopic scale on the following positions: lipids on 0.9, 2.3, 2.8, and 5.2 ppm, water at 4.7 ppm, and Cho at 3.2 ppm. The presence of elevated Cho peak was classified according to binary classification: present/absent.

Two breast-dedicated radiologists analyzed the imaging data in consensus (N.P.B. with 15 years of experience in breast imaging and J.B. with 11 years of experience in breast imaging); a magnetic resonance engineer (O.S.) experienced in postprocessing of spectroscopic data (17 years of experience) was involved in obtaining, processing, and analyzing of the spectra.

### Histological Analysis

Histological examination was performed on the samples obtained on percutaneous, core biopsy, or surgical open excision. Pathohistologic findings provided information on the lesion type (benign/malignant) and histological diagnosis based on the WHO Classification of the breast tumors and the Classification of the benign lesion of the breast (9). Hormone receptor status was determined using immunohistochemistry analysis while Her2 (human epidermal growth factor receptor 2) status was defined using *in situ* hybridization.

### Statistical Analysis

Statistical analysis was performed using SPSS software package for Windows, ver. 19.0 (IBM, Chicago, IL, USA). The sample was explored using descriptive and comparative methods. Descriptive statistics was used to define mean, median, minimum, and maximum values, interquartile range, and standard deviation, depending on the type of the variable. For hypothesis testing, we used *χ* ^2^ test. To test the level of agreement between two different methods, we used Cohen’s Kappa test (K value ranges between 0 and 1, depending on the level of agreement: <0.2, poor; 0.21–0.4, fair; 0.41–0.6, moderate; 0.61–0.8, good; and >0.81, very good). Receiver operating characteristic (ROC) curves were constructed to determine sensitivity, specificity, and positive and negative predictive vaules, using standard formulas. Statistical significance was set at value *p* < 0.05.

## Results

**Figure 1:**
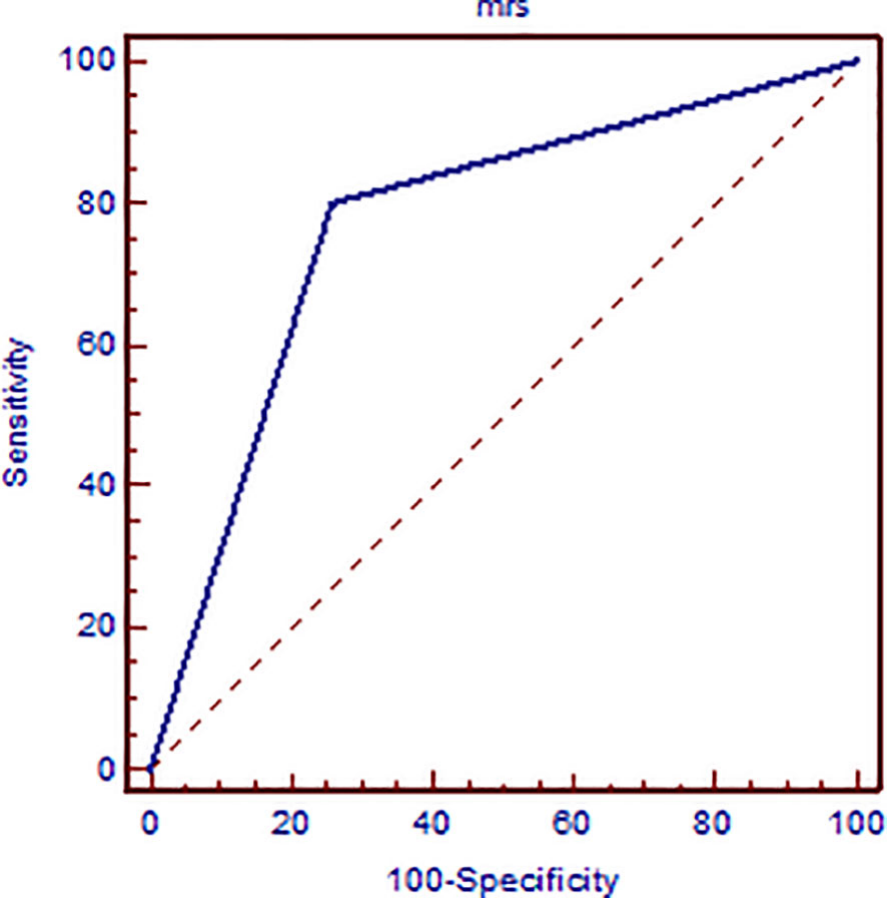
ROC curve analysis presenting the sensitivity and specificity of elevated Cho peak in detecting breast cancer malignancy confirmed on histologic examination.

A total of 57 lesions were detected and analyzed on ^1^H-MRS: 30 malignant and 27 benign lesions. The BI-RADS classification results of MR of the breast in relation to histological and MRS findings are presented in **Table 2**. Elevation of the Cho peak was detected in 24/30 malignant lesions (80.0%), while in 6 lesions (0.2%), it was not observed. It was also present in 7/27 benign lesions (25.93%). Majority of benign lesions (20/27, 74.07%) did not exhibit elevation of the Cho peak. Sensitivity of the method was 0.8, specificity was 0.741, positive predictive value was 0.774, and negative predictive value was 0.769. Kappa test showed only moderate agreement between ^1^H-MRS and histologic findings (0.542). In **Figure 1**, the results of ROC curve analysis are shown—area under the curve is 0.770 [CI (confidence interval) 0.640–0.871].

**Table 2:** The results of BI-RADS classification and the presence of Cho peak on the magnetic resonance spectroscopy.

BI-RADS category	Histologically benign	Histologically malignant	Cho peak present	Cho peak absent
**3—probably benign**	17	0	2	15
**4—suspicious**	9	3	8	4
**5—highly suspicious**	1	27	22	6
**Total**	27	30	32	25

Mean size of the malignant lesions was 35 mm, and that of benign lesions was 20 mm. Malignant lesions that showed elevation of the Cho peak were significantly bigger (39.4 mm, 18–70 mm) than those without Cho peak (22.6 mm, 12–35 mm) (*p* < 0.01). A similar situation was observed in benign tumors: lesions with elevated Cho peak were bigger (24.5 mm) than those without elevation of Cho peak (18.3 mm), but it did not reach significance (*p* > 0.05).

The distribution of the histologic type of the breast cancer with elevated Cho peak was as follows: invasive ductal cancer (DCI) was the most frequent (90.5% of malignant lesions with elevated Cho were DCIs, 12.5% lobular cancer, 4.2% DCIS, and 4.2% medullary cancer), while in benign lesions, 71.4% with elevated Cho were adenoid fibroadenomas, 14.3% hamartomas, and 14.3% trichofolliculoma.

Sensitivity of MRM in this study population was 100%, while specificity was 90.9%.

## Discussion

Most studies to date have aimed to determine if the presence of tCho resonance can be an indicator of lesion malignancy, based on the detection of intracellular phosphocholine in the malignant lesion of the breast (5).

**Figure 2:**
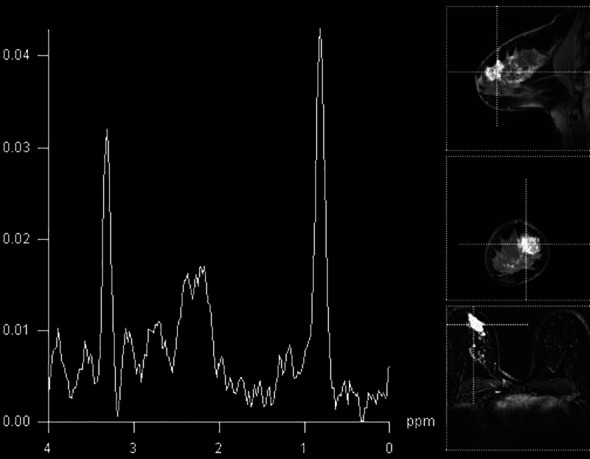
Breast MR spectroscopy in the BI-RADS 5 lesion in the right breast (histologically confirmed lobular cancer) with elevated Cho peak on 3.2 ppm.

In our study population, the detection of elevated Cho peak was shown to be a good indicator of breast lesion malignancy (**Figure 2**), still not overweighing the sensitivity or specificity of DCE-MRI. The sensitivity of the method was good, 0.8, while that of DCE-MRI reached 100%, similar to recent studies (10, 11). The specificity of ^1^H-MRS was 0.741, while that of DCE-MRI was 0.909. The agreement between two methods was only moderate. A recent study by Cavedon et al. showed that inclusion of MRS increased sensitivity and specificity of support-vector-machine analysis from 93.7% and 84.9% to 95.1% and 90.7%, respectively, in differentiation between malignant and benign lesions (12).

In our opinion, there are several explanations that contributed to these findings and have been separately addressed. Our study concluded that lesion size represents an important feature of the breast lesion, showing that malignant tumors with positive Cho peak were significantly bigger than Cho-negative ones. The difference in size in benign lesions regarding the presence of Cho peak did not reach significance, although Cho-positive lesions were bigger. Similar results were obtained in the previous studies (13–15). According to these authors, MRS seems to be limited in the lesions smaller than 20 mm, which makes it unreliable in the early stage of disease (T1 stage). However, these studies were based on a single-voxel technique and on clinical scanners of lower field strength. High- and ultrahigh-field scanners (≥3 T) might be able to overcome these limitations, since the ability of detecting low concentrations of metabolites is significantly improved in modern units. Indeed, Katz-Brull et al. reported that the sensitivity of detecting tCho increased steadily with the increase in tumor size (16). Additionally, it has already been shown that smaller lesions exhibited Cho peaks more consistently on 3 T than on 1.5 T (17, 18).

There are some points regarding the voxel size that also need to be mentioned. The voxel size in our study was 1 ml. Voxel volumes vary greatly across the studies, since currently there are no established evidence-based recommendation. It is a known fact that larger voxel has a better signal-to-noise ratio, and therefore, the detection of the presence of Cho should be easier. The additional concern is the concentration of tCho in the voxel that affects the detection greatly. Meta-analysis showed that a voxel concentration of Cho of 1.45 mmol/kg has a sensitivity of 0.732 and a specificity of 0.767, and that a concentration of 1 mmol/kg could be detected in only about 50% of the cases on 1.5 T (19). It must be accentuated that these studies, as well as our own study, did not include a typical high-risk screening population but women with symptomatic and/or palpable breast lesions. The most recent study by Sodano et al. confirmed that a cutoff of 0.8 mmol/L tCho can determine the malignant nature with a sensitivity of >95% (20). This study pointed to the valuable potential of multiparametric MRI using MRS as an adjunct tool to downgrade suspicious breast lesions, as well as to diagnose malignancy spreading to regional lymph nodes (20).

**Figure 3:**
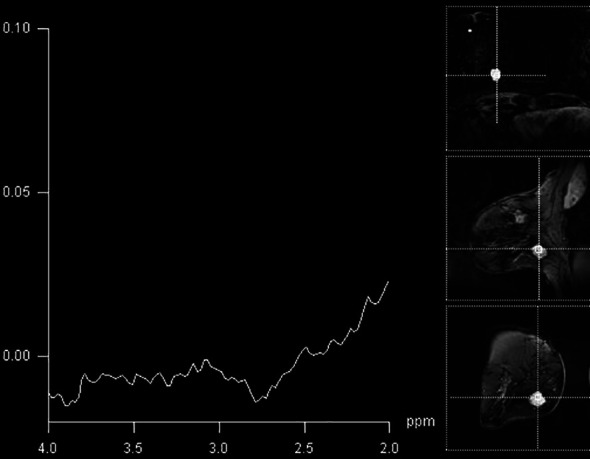
A false-negative finding on MR spectroscopy of BI-RADS 4 lesion in the breast (histologically confirmed medullary cancer), with absent Cho peak.

In our study, it was noticed that the detection of elevated Cho peak was dependent on the type of tumor. The presence of Cho peak was observed in 90.5% of ductal invasive cancers (19/21), while it was detectable in only 50% of invasive lobular cancers (3/6) (**Figure 3**). The only case of mucinous breast cancer that was included in the study did not show the presence of choline peak, while medullary cancers all showed elevation of this marker. Baek et al. showed higher sensitivity and specificity in mass compared to non-mass lesions, but did not go further into separating tumors according to histological diagnosis (21). Montemezzi et al., on the other hand, in a recent study using multiparametric MRI on 453 lesions, showed that Cho peak was consistently elevated in triple-negative cancers, compared to Luminal-A and Luminal-B types (22). However, it must be noted that triple-negative tumors in this study were also significantly higher than other subtypes. This study concluded that different techniques in multiparametric imaging showed strengths in detection of different subtypes of tumors, and that no specific technique has high enough specificity when interpreted alone. Sharma et al. analyzed MRS in breast cancers with indeterminate DCE-MRI findings and showed that the sensitivity of MRS was 89.3%, and that the additional value of MRS was lower than that of DWI in the same population (23). However, this study only included malignant lesions and the aim was to clarify the indeterminate findings of DCE-MRI with the additional advanced imaging tools.

**Figure 4:**
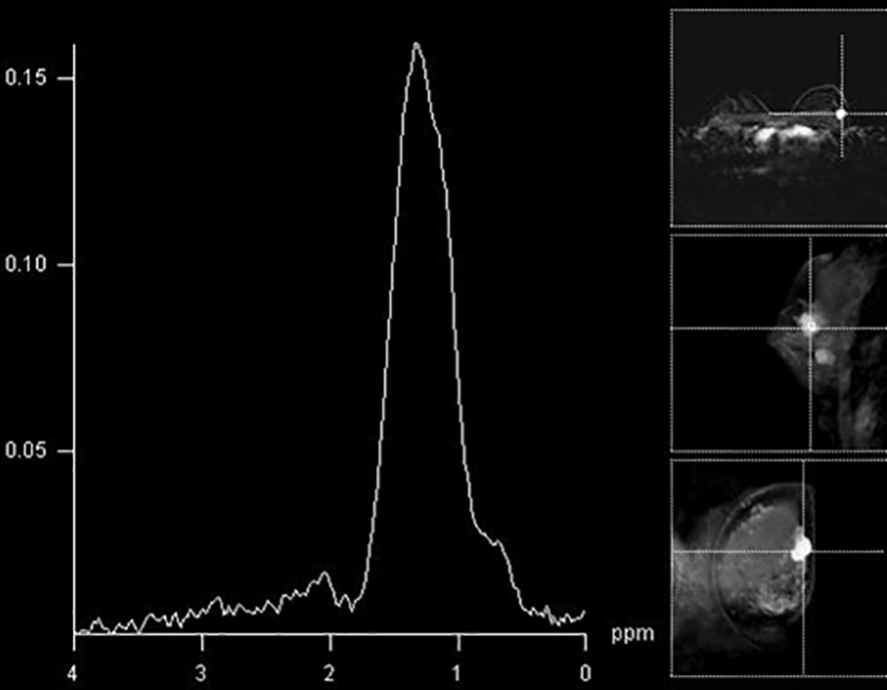
The absence of a Cho peak in a histologically benign breast lesion located in the right breast (fibroadenoma).

**Figure 5:**
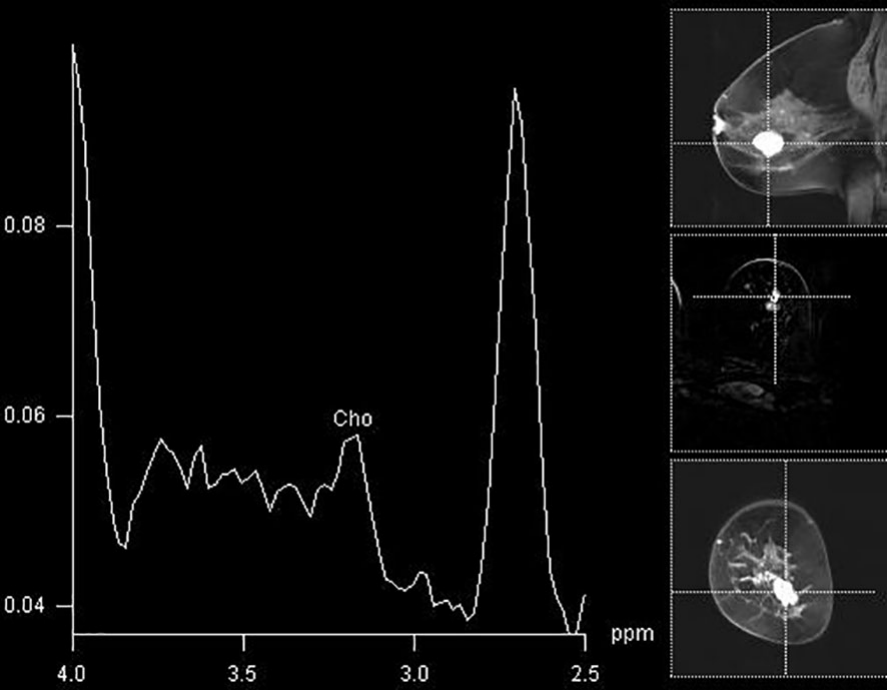
A false-positive finding of an elevated Cho peak in the lower lateral aspect of the right breast in a histologically verified hamartoma.

Most of the benign tumors did not present with Cho peaks (**Figure 4**). Interestingly, some benign tumors exhibited the presence of Cho peak (**Figure 5**). Most of these lesions were adenoid fibroadenomas and were slightly bigger in size than Cho-negative benign tumors. Given that adenoid fibroadenomas often present with Type II curve on DCE-MRI, it is important to be cautious when interpreting MRS findings in these lesions.

We failed to present correlations of histological grade of the tumor with the presence of Cho peak, but, in our opinion, it should be attributed to a small study sample. Additionally, a recently raised question of intratumoral heterogeneity might be interesting. However, the use of multivoxel spectroscopy should overcome this obstacle, especially bearing in mind that intertumoral differences are greater than intratumoral ones, so one representative spectrum (if adequately chosen) could be enough for determination of malignancy of breast lesion (24).

### Limitations

There are some limitations of the study. First, we only included the elevation of the Cho peak in the analysis, without considering other peaks evident on MRS, such as lipid peaks. The inclusion of additional peaks could improve specificity of the findings, as shown by Thakur et al. (25), who presented reduction in several lipid peaks in malignant lesions, compared to benign ones.

Secondly, we only analyzed spectra in the region of the tumor with the most intensive contrast enhancement. Inclusion of the spectra in the surroundings of the lesion could point to the invasive nature of the lesions, similarly to the findings in the brain (26, 27).

Finally, even though several techniques are recommended for performing MRS in the breast lesions (28), current opinion is that the technique itself could be improved, regarding spectra acquisition and interpretation as well as study design and patient recruitment (29). Additionally, in the means of the study design, it might be wise to estimate the Cho peak in the breast lesions before and after contrast administration to evaluate the effect of neutral gadolinium chelates on the MR spectra and to avoid possible interfering of metal chelate with the Cho peak.

## Conclusion

Although elevation of the choline peak has a good sensitivity and a satisfying specificity in breast cancer detection, both are significantly lower than those of multiparametric MRM. Inclusion of spectra located in the tumor surrounding tissue as well as analysis of lipid peaks could aid both sensitivity and specificity. An important ratio of false-positive and false-negative findings in specific types of breast lesions (lobular cancer and adenoid fibroadenoma) suggests that these lesions should be interpreted with a reasonable level of caution.

## Data Availability Statement

The raw data supporting the conclusions of this article will be made available by the authors, without undue reservation.

## Ethics Statement

The studies involving human participants were reviewed and approved by the Ethical Committee of Faculty of Medicine, University of Novi Sad. The patients/participants provided their written informed consent to participate in this study.

## Author Contributions

NP and JB drafted the manuscript. OS and NP performed the analysis, collected the data, and performed statistical analysis. DK participated in data analysis and interpretation. JB revised the manuscript for the intellectual content. All authors contributed to the article and approved the submitted version.

## Funding

The funding for this study was partially obtained from the H2020—A multimodal AI-based toolbox and an interoperable health imaging repository for the empowerment of imaging analysis related to the diagnosis, prediction and follow-up of cancer—INCISIVE, No. 952179.

## Conflict of Interest

The authors declare that the research was conducted in the absence of any commercial or financial relationships that could be construed as a potential conflict of interest.

## Publisher’s Note

All claims expressed in this article are solely those of the authors and do not necessarily represent those of their affiliated organizations, or those of the publisher, the editors and the reviewers. Any product that may be evaluated in this article, or claim that may be made by its manufacturer, is not guaranteed or endorsed by the publisher.
